# Why Do Drivers Use Mobile Phones While Driving? The Contribution of Compensatory Beliefs

**DOI:** 10.1371/journal.pone.0160288

**Published:** 2016-08-05

**Authors:** Ronggang Zhou, Mengli Yu, Xinyi Wang

**Affiliations:** School of Economics and Management, Beihang University, Beijing, P. R. China; University of Akron, UNITED STATES

## Abstract

The current study is the first to investigate the contribution of compensatory beliefs (i.e., the belief that the negative effects of an unsafe behavior can be "neutralized" by engaging in another safe behavior; e.g., "I can use a mobile phone now because I will slow down ") on drivers’ mobile phone use while driving. The effects of drivers’ personal characteristics on compensatory beliefs, mobile phone use and self-regulatory behaviors were also examined. A series of questions were administered to drivers, which included (1) personal measures, (2) scales that measured compensatory beliefs generally in substance use and with regard to driving safety, and (3) questions to measure drivers’ previous primary mobile phone usage and corresponding self-regulatory actions. Overall, drivers reported a low likelihood of compensatory beliefs, prior mobile phone use, and a strong frequency of self-regulatory behaviors. Respondents who had a higher tendency toward compensatory beliefs reported more incidents or crash involvement caused by making or answering calls and sending or reading messages. The findings provide strong support for the contribution of compensatory beliefs in predicting mobile phone usage in the context of driving. Compensatory beliefs can explain 41% and 43% of the variance in the active activities of making calls and texting/sending messages compared with 18% and 31% of the variance in the passive activities of answering calls and reading messages. Among the regression models for predicting self-regulatory behaviors at the tactical or operational level, compensatory beliefs emerge as significant predictors only in predicting shorter conversations while on a call. The findings and limitations of the current study are discussed.

## Introduction

Driver distraction can be defined as the diversion of attention away from activities critical for safe driving and toward a competing activity [1]. Mobile phone use is one of the most serious driving distractions, and its negative influence on driving performance has been supported by a large body of recent research [2, 3–9]. Mobile phone use in conjunction with more physical demands, such as using a handheld phone, making a call, and sending a short message, tends to be perceived as riskier. Drivers tend to give negative responses when asked about their intention to use a phone while driving. However, mobile phone use while driving represents a pervasive worldwide phenomenon, and a large number of drivers use their phones while driving. For example, in 2014, among all road incidents in China (656.3 ten thousands), 47.2% were caused by mobile phone use when driving, which was the most distracting activity (The Traffic Management Bureau of the Ministry of Public Security of China, 2014). These statistics lead to a question: when a driver knows that using a mobile phone while driving is risky but needs to use a mobile phone while driving, how does the driver modulate his/her behavior accordingly? The fundamental question regarding the effect of competing activities on driver performance or driving safety is whether and how drivers compensate for any decrease or riskiness in attention to the driving task to maintain adequate safety margins. Surprisingly, little research has directly addressed this problem [10]. Given the safety implications of mobile phone use while driving and the scarcity of studies focusing on drivers’ compensatory beliefs(e.g., general compensatory beliefs, such as “smoking can be compensated for by exercising”, and safe compensatory beliefs, such as “I can make a call now because I will shorten the conversation while driving”), this study aimed to consider the contributions of drivers’ compensatory beliefs in exploring why people use mobile phones while driving and the corresponding self-regulatory actions.

### Typical distracting activities involved in mobile phone use while driving

Self-regulatory behaviors are motivated by distracting activities in the context of driving, so it is necessary to understand the typical usage of mobile phones while driving. Some current studies suggest that the order of frequency for the most common mobile phone activities while driving is (1) answering calls, (2) making calls, (3) reading messages, and (4) texting and sending messages [11–16]. The behavioral decision to engage in these types of cell phone usage are related to drivers’ perceptions of the risk of these activities [12, 13, 17, 18]. When drivers were asked to rate the perceived degree of risk for distracting activities, the rank order from most to least dangerous was texting and sending messages, reading messages, dialing a mobile phone, and answering a mobile phone [15, 19]. With epidemiological method, the rate of accidents caused by cell phone use while driving can be calculated as relative actual risk, and send text message was also ranked as the most dangerous use among all distractive activities causing by cell phone use (odds ratio = 23), following by dialing call (2.8–5.9), reading messages (odds ratio = 3.4–4.0), and answering calls (odds ratio = 1.4–3.1) [20, 21]. As shown by the previous studies mentioned above, the rankings of the four typical types of cell phone usage in terms of engagement frequency, perceived risk or degree of distraction and actual level of risk were consistent. However, a "perfect" risk perception of cell phone usage cannot effectively curb the actual behavior. This may be because drivers tend to adopt self-regulatory behaviors to compensate for the possible risk according to their perceptions of the degree of distraction or risk.

Overall, the collective results of these studies suggest that making/answering calls and texting/reading messages are pervasive in the context of mobile phone use while driving, and people’s decisions to use self-regulatory actions to keep their driving within an adequate safety margins vary with the degree of engagement in these typical distracting activities. It is therefore important to focus on these common types of cell phone usage in our attempt to examine why drivers use mobile phones while driving from the perspective of self-regulatory or compensatory actions.

### Engagement in self-regulatory behaviors for cell phone use while driving

To focus on this issue, it is important to determine whether and how drivers self-regulate their driving to compensate for the impairment caused by phone use. In research documenting the effect of mobile phone use on driving safety, attention has recently been given to the possible compensatory behaviors involved in mobile phone use while driving, including stopping the vehicle [22], reducing speed or slowing down [5, 23], increasing headway distance [3, 24, 25], limiting the amount of lane changing [26], and paying more attention to the road situation [27]. However, collective evidence from the majority of current studies does not indicate compensation or its opposite [28, 29]. Some findings indicate that drivers may reduce their speed and increase their following distance in response to changing or competing task demands to maintain an adequate level of safe driving [5, 23, 25, 30]. Drivers may compensate for the deleterious effects of cell phone use by decreasing their speed when using a handheld phone but may neglect to do so when using a hands-free phone [31]. However, some researchers have found that drivers do not compensate for impairment during cell phone conversations by increasing headway or decreasing their speed during the phone task [32, 33]. Furthermore, drivers who use either phone type (i.e., handheld or hands-free mobile phones) do not appreciably compensate by increasing following distance or reducing speed [28]. Based on simulated driving, these studies cannot resolve the controversial point of whether self-regulatory actions can be interpreted as changes in driving behavior due to loss of control [34] or whether they can be viewed as examples of a performance trade-off due to assignments to competing activities [10].

Some studies using either self-report or observational methods to examine on whether drivers take initiative to engage in self-regulatory actions to compensate for impairments in driving performance may address this contradictory issue. Other types of compensatory behaviors that are more directly related to mobile phone use (such as shortening the conversation time while on a mobile phone and reminding the caller that the driver is currently driving) have also been considered in this line of research [18, 19, 35]. For example, Young and Lenné found that 75% of drivers reported modifying their driving in a number of ways when they engaged in other tasks while driving [19]. These authors found that the most common behaviors engaged in were reducing speed (78.2%), pulling over to the side of the road (67.8%), increasing following distance from the car ahead (48.0%) and stopping the vehicle (42.0%). The duration of phone calls while driving is typically short, with 61% of drivers reporting that their average phone call lasts 1 minute or less [19]. In a sample of licensed teenage drivers, O’Brien et al found that teens also reported using several strategies to reduce the risk associated with cell phone use while driving: more than 80% of teens who had ever talked on a cell phone while driving said that they attempted to keep their conversations short because they were driving, and among teens who had ever texted while driving, approximately half said that they often waited until they felt safe to read and reply to text messages (58% and 47%, respectively) [35]. Zhou et al.’s research indicated that more than 90% of drivers said they were likely to engage in compensatory actions by reminding the caller that they were driving and limiting the conversation in both scenarios, and they reported that conversations should be shorter in the handheld scenario (0.81 min) than in the hands-free scenario (1.38 min) [18]. All of these studies suggest that drivers can take the initiative to use self-regulatory behaviors related to driving or conversation to compensate for impaired driving performance. In accordance with Young et al.’s findings in [10], drivers may engage in self-regulatory actions at strategic, tactical, or operational levels when driving.

Discussions are ongoing regarding whether behavioral changes involve conscious regulations, indications of a loss of control or both [34]. Previous studies have typically used driving simulators and questionnaire surveys and have collectively found that we can attempt to explain why drivers frequently use mobile phones while driving from the perspective of drivers’ self-regulatory actions.

### Drivers’ compensatory beliefs and cell phone use while driving

The collective findings using the Theory of Planned Behavior (TPB) [36] indicate that drivers’ frequency of mobile phone use while driving can be explained by drivers’ beliefs regarding behaviors, norms, and personal ability [13, 18, 37–39] proposed a structural model of compensatory intentions modified from the TPB to explain drivers’ engagement in self-regulatory behavioral decisions and found that the decision to answer an incoming call and perceived behavioral risk control could consistently explain most of the variance in both handheld and hands-free use for the compensatory perceptions of conversation limits. However, all previous efforts to explain why drivers use mobile phones while driving have overlooked the possible effects of drivers’ compensatory beliefs. According to findings in the health domain [40–43], compensatory health beliefs involve the belief that the negative effects of an unhealthy behavior can be compensated for or "neutralized" by engaging in another healthy behavior (e.g., "I can eat this piece of cake now because I will exercise this evening") [44], and the activation of these beliefs result in failure to achieve health goals (e.g., to maintain one’s figure or control body weight). In line with this definition, compensatory green beliefs, which refer to beliefs that the negative effects of energy-inefficient or unsustainable behaviors (e.g., flying abroad for vacation) can be compensated for by engaging in energy-efficient or sustainable practices (e.g., using public transport), was also scaled and documented as a significant variable for predicting environmentally important behaviors [45, 46]. With respect to mobile phone use when driving, drivers may hold two types of behavioral beliefs (i.e., "mobile use will negatively affect driving safety" or "shortening conversations or slowing down can minimize the impairment to driving performance due to mobile phone use"). These two types of beliefs can be activated jointly to trigger a compensatory safe belief, such as "I can use a mobile phone now because I will slow down". The degree of actual engagement with mobile phones while driving partially depends on whether an individual holds this compensatory belief. However, in the field of driving safety, no study has addressed drivers’ general compensatory beliefs or compensatory beliefs regarding driving or tested the possible effects of these beliefs on risky or distracted driving, such as cell phone use while driving.

### The aims of the current study

As motioned above, increasing numbers of studies have addressed the influence of compensatory beliefs on human actions or behavior in the health or green environmental domains. These studies have found that compensatory beliefs are a strong and stable predictor of unsafe or unhealthy behaviors. However, to date, no studies have investigated drivers’ compensatory beliefs or tested how these beliefs predict mobile phone use in the context of driving. The current study is the first to consider drivers’ compensatory beliefs within the field of driving safety to explore why people use mobile phones while driving. In this study, we extend the findings of studies on healthy and green compensatory beliefs to examine drivers’ distraction due to engagement in mobile phone uses. The four typical uses of mobile phones, making calls, answering calls, texting and sending messages, and reading messages, were selected as the four main types of mobile use while driving. The current study uses both indirect and direct scales to measure respondents’ compensatory beliefs and then addresses the relationship between compensatory beliefs and cell phone use while driving. Thus, the main goals of this study were as follows: (1) to assess drivers’ compensatory beliefs and address the frequency of engagement of different self-regulatory actions for mobile calls or messaging and to examine the effects of basic demographic measures (gender, age, and driving experience) on drivers’ compensatory beliefs, prior mobile phone use and corresponding self-regulatory behaviors; (2) to address whether mobile phone usage while driving can be predicted by drivers’ compensatory beliefs and to investigate the contributing effects of compensatory beliefs and mobile use to predict corresponding self-regulatory behaviors.

## Method

### Participants

The study protocol was approved by the Human Research Ethics Committee in School of Economics and Management at Beihang University. The survey was conducted by an online questionnaire. We asked a local professional market survey firm to invite drivers to answer the online questionnaire. A total of 213 drivers participated in this survey. To ensure that the responses were reliable, some general criteria were used for the selection of valid responses, as follows: (1) the same scores were used for most or all scaled items; and (2) one or more items were not answered. Of those who met the requirements, two respondents reported that their driving frequency was less than one day per week. Thus, as shown in Table 1, a total of 140 respondents’ answers were used in this study. Of these respondents, 60% were male and 40% were female. With respect to age group, 10% were aged 21–25 years, 35.7% were aged 26–30 years, 42.1% were aged 31–40 years, 10% were aged 41–50 years, and 2.1% were aged 51–60 years. Thus, 45.7% of the participants were in the younger group (21–30), and 54.3% were in the older group (30–60). In terms of educational level, 79.3% of the participants had bachelor’s degrees, 11.4% had less education (i.e., associate, secondary technical school, high or middle school or below), and 9.3% had master’s degrees or above. With respect to driving patterns or experience, 48.6% of all respondents had driving experience of two to five years, whereas the rest had six or more years of driving experience. Most of the respondents (79.3%) reported driving frequency of at least four days per week. Furthermore, 19.3% and 20% of the respondents reported that they had experienced accidents or incidents caused by calls or messaging, respectively. In addition, the mean driving mileage per year was 12376.5 kilometers, and the mode was 10,000 kilometers.

**Table 1:** Respondents’ demographic profiles and driving patterns.

Measures	Frequency	Percentage
Age group		
21–30	64	45.7
31–60	76	54.3
Gender		
Male	84	60.0
Female	56	40.0
Education		
Associate or below	16	11.4
Bachelor	111	79.3
Master or above	13	9.3
Driving age (years)		
2–5	68	48.6
6 or above	72	51.4
Driving frequency per week		
One day	4	2.9
Two days	7	5.0
Three days	18	12.9
Four days	19	13.6
Five days	42	30.0
Six days	29	20.7
Seven days	21	15.0
Prior incidents or accidents report		
Caused by making or answering calls	27	19.3
Caused by sending or reading messages	28	20.0

The respondents were ensured that their participation was voluntary and that they would be remunerated for their anonymous responses. Before participants answering the questions, the written background information regarding the survey was introduced in the first page, and potential respondents knew that they would not be punished if they chose not to participate. The voluntary behavior of answering the questionnaire was thought of as agreement to participate in the research. It took the respondents approximately five minutes to complete the survey.

### Questionnaire measures

The survey contained four sections. The first two sections were the primary sections of the survey and contained the same questions about general compensatory beliefs and compensatory safe belief variables. The next section collected the respondents’ prior mobile phone usage behaviors and corresponding self-regulatory behavior for mobile use in the context of driving. The final section was developed to establish the demographic profile. The details of the questionnaires are presented below.

#### General compensatory beliefs of substance use

At the beginning of the study, we attempted to use the compensatory health scale to measure drivers’ compensatory beliefs [40]. Before the questionnaire could be used in the Chinese context, it had to be translated and its psychometric properties had to be tested with principal component analysis (PCA) and internal consistency analysis. Therefore, prior to the main study, we translated the questionnaire in a pilot study in accordance with guidelines for cross-cultural adaptation and tested it in a sample of Chinese adults (M = 29.8 years, SD = 7.89 years and aged 20–68 years; 78 (49.7%) were males and 85 (52.1%) were females; 3.7% (n = 6) had high school education, 50.3 (n = 82) had mid-level education with an associate’s degree, 43.6% (n = 71) had a bachelor’s degree, and 2.5% (n = 4) held a master’s degree). However, as shown in the Dutch context, the original Canadian scale was not supported in the Chinese context. The PCA procedure did not yield a four-component solution with corresponding items categorized in the subscales of substance, eating/sleeping habits, stress, and weight regulation. Thus, the responses for the questionnaire items in the four original subscales were submitted separately to a PCA: substance use accounted for 51.45% of the variance (Cronbach’s α 0.80), stress accounted for 56.47% of the variance (Cronbach’s α 0.74), eating/sleeping habits accounted for 56.02% of the variance (Cronbach’s α 0.71), and three items in weight regulation yielded more than one component. Consistent with previous studies [40, 47], the internal consistency of the subscale of substance use was higher than the others, potentially indicating stability. In addition, the compensatory healthy scale was used to measure healthy or unhealthy activities. The subscales of stress and eating/sleeping are targeted toward the measurement of compensatory health beliefs more than substance use. Based on this consideration, the subscale of substance use was used to measure drivers’ common compensatory beliefs in the main study.

The six items to measure substance use in the compensatory health scale were “Smoking from time to time is OK if one eats healthy”, “Exercising can compensate for smoking”, “Eating healthy can make up for the effects of regularly drinking alcohol”, “It is alright to drink a lot of alcohol as long as one drinks lots of water to flush it”, “Not drinking alcohol during the week can make up for the effects of drinking too much alcohol during the weekend”, and “The effects of drinking coffee can be balanced by drinking equal amounts of water” [40]. All statements were rated by respondents on a seven-point scale from 1 (*strongly disagree*) to 7 (*strongly agree*). The data of the driver respondents on substance use were submitted to a PCA. With use of an eigenvalue greater than 1 as a criterion (i.e., identifying a clear elbow point with an eigenvalue greater than 1.0 in a scree test), the PCA procedure yielded a single-factor solution that accounted for 68.59% of the variance. The factor loading for response items ranged from 0.71 to 0.88. In addition, internal consistency analysis was conducted to check the reliability of the questionnaire, and the Cronbach’s α statistic was 0.91. These psychometric analyses showed that the self-reports of compensatory beliefs used this study were valid and reliable.

#### Compensatory safety beliefs toward mobile use while driving

Two items were used to measure respondents’ perceived safe compensatory beliefs toward mobile use in driving situations: “When answering a call in a driving situation, using a hands-free mobile phone is safer than using a handheld mobile phone” and “When answering a call in a driving situation, reducing speed can make up for driving safety”. The statement was rated by the respondents on a seven-point scale from 1 (*strongly disagree*) to 7 (*strongly agree*). The PCA identified a single component that accounted for 70.44% of the variance (α = 0.58).

#### Prior mobile usage and corresponding self-regulatory behaviors in a driving context

Based on a combination of the research aims and previous studies [19], a total of fifteen questions were used to measure drivers’ prior mobile usage and corresponding self-regulatory behaviors in driving situations, including call-related activities and short message-related activities along with corresponding reports of the frequency of self-regulatory driving or mobile use behaviors. With respect to call-related usage, the respondents were first asked how frequently they made calls and answered calls. Then, they were asked to rate their behavioral frequency of slowing down, pulling over to the side of the road, increasing distance from the car ahead, changing lanes less frequently, shortening conversations, reminding the caller that the driver is driving, and refusing to answer calls when using mobile phones to make or answer calls in driving situations. In terms of short message-related usage, after the respondents answered how frequently they sent short messages and read short messages while driving, four items were used to measure the behavioral frequency of slowing down, pulling over to the side of the road, increasing distance from the car ahead, and changing lanes less frequently when reading short messages in driving situations. These questions were presented following the questions on demographic and driving pattern measures. Each item was rated on a seven-point scale ranging from 1 (*very infrequently*) to 7 (*very frequently*).

#### Demographic measures and driving experience measures

Demographic information including age group, gender, and education was gathered. Information was also gathered on driving patterns or experience in relation to driving age, number of miles driven, driving frequency per week, and prior incidents or accidents experienced due to mobile use while making or answering calls and sending or reading short messages.

## Results

The data were analyzed using SPSS version 19.0. For behavioral intentions or decisions, measures of perceived behavioral control and perceived risks, and activities of mobile phone use, we first calculated descriptive statistics in percentages and average response scores. Table 2 presents these results for the main study variables. Then, we used an analysis of variance (ANOVA) to test for differences in the average response scores for drivers’ compensatory beliefs, engagement in prior mobile use and its self-regulatory behaviors in driving contexts. Finally, linear regression was used to analyze the contributions of compensatory beliefs for predicting prior mobile usage and corresponding prefigured self-regulatory predictors. The results of zero-order correlations and a series of multiple regression analyses are shown in Tables 2, 3 and 4.

**Table 2:** Descriptive statistics and zero-order correlations between the study variables.

Measures	1	2	3	4	5	6	7	8	9	10	11	12	13	14	15	16	Lower3	Higher3	M	S.D
1. compensatory beliefs	_																65.71	8.57	2.75	1.28
2. safe compensatory beliefs	.69^a^	_															47.14	17.14	3.14	1.42
3. make call	.67^a^	.58^a^	_														66.43	23.57	2.92	1.56
4. answer call	.45^a^	.36^a^	.72^a^	_													37.86	40.00	3.92	1.55
5. slow down while making/answering calls	-.11	.01	-.01	.12	_												6.43	86.43	5.55	1.24
6. pull over when making/answering calls	.17^c^	.03	-.01	-.08	-.05	_											26.43	60.00	4.51	1.57
7. increase distance when making/answering calls	-.07	-.01	-.05	.05	.39^a^	.21^c^	_										4.29	83.57	5.44	1.13
8. change lanes less frequently while making/answering calls	-.06	-.03	.03	-.01	.34^a^	.09	.46^a^	_									8.57	77.14	5.28	1.42
9. shorten conversation while making/answering calls	-.36^a^	-.31^a^	-.22^b^	-.03	.29^b^	.05	.45^a^	.32^a^	_								3.57	89.29	5.75	1.10
10. remind the caller that he/she is driving	-.24^b^	-.22^c^	-.20^b^	-.10	.19^c^	.20^c^	.27^b^	.14	.52^a^	_							3.57	91.43	5.81	1.10
11. refuse to answer call	.03	.03	-.07	-.26^b^	-.18^c^	.34^a^	.02	-.02	-.01	.15	_						31.43	44.29	4.28	1.46
12. send short message	.67^a^	.54^a^	.62^a^	.36^a^	-.14	.11	-.05	.01	-.30^a^	-.20^c^	.14	_					70.00	17.14	2.51	1.68
13. read short message	.52^a^	.50^a^	.60^a^	.46^a^	-.01	.07	.13	.17^c^	-.06	-.10	.03	.76^a^	_				55.71	29.29	3.24	1.76
14. slow down when reading message	.00	.13	.08	.18^c^	.44^a^	.00	.40^a^	.45^a^	.36^a^	.11	-.09	.04	.23^b^	_			13.57	77.14	5.12	1.58
15. pull over when reading message	.25^b^	.15	.13	.04	.06	.51^a^	.06	.07	-.01	.02	.26^b^	.15	.13	.37	_		34.29	51.43	4.25	1.85
16. increase distance when reading message	.01	.13	.03	.15	.45^a^	.03	.42^a^	.41^a^	.36^a^	.08	-.03	-.04	.17^c^	.84^a^	.52^a^	_	12.86	74.29	5.07	1.55
17. change lanes less frequently when reading message	.12	.15	.14	.22^b^	.41^a^	.07	.40^a^	.48^a^	.22^b^	.09	-.10	.05	.27^b^	.73^a^	.40^a^	.73^a^	19.29	70.71	4.88	1.65

Pull over means pull over to side of road; increase distance means increase distance from car ahead.

^a^ *p* < 0.001;

^b^ *p* < 0.01;

^c^ *p* < 0.05.

**Table 3:** Hierarchical regression analysis: predicting mobile phone usage while driving.

Steps and predictors	Make call (β)	Answer call (β)	Send message (β)	Read message (β)
1. age group	-.24**	-.18*	-.19*	-.14
gender	-.07	.07	-.11	-.11
driving age	.14	.15	-.03	-.07
*R*^2^	.057	.048	.043	.036
*F* (3, 136)	2.76*	2.31	2.03	1.68
2. age group	-.13	-.11	-.09	-.05
gender	-.04	.09	-.08	-.08
driving age	.06	.10	-.11	-.15
safe compensatory beliefs	.55***	.33***	.54***	.51***
*R*^2^	.350	.153	.319	.284
△*R*^2^	.293	.105	.276	.248
*F*_change_ (1, 135)	60.80***	16.70***	54.71***	46.77***
3. age group	-.03	-.04	.03	.03
gender	-.01	.11	-.05	-.07
driving age	.03	.08	-.15*	-.17*
safe compensatory beliefs	.22*	.08	.16	.28**
general compensatory beliefs	.50***	.38**	.57***	.35**
*R*^2^	.471	.223	.477	.342
△*R*^2^	.121	.070	.158	.058
*F*_change_ (1, 134)	30.55***	12.00**	40.54***	11.74**

* *p* < 0.001;

** *p* < 0.01;

*** *p* < 0.05.

**Table 4:** Regression analysis: predicting self-regulatory behaviors.

Self-regulatory behaviors	*R*^2^	*F*	Age group	Gender	Driving age	Active behavior	Passive behavior	CB	SCB
1. slow down while making/answering calls	.07	1.35	.02	.05	.05	-.10	.24	-.26	.17
2. pull over when making/answering calls	.08	1.52	-.04	-.06	.04	-.09	-.14	.36 ^b^	-.13
3. increase distance when making/answering calls	.03	0.52	-.05	-.05	.00	-.17	.19	-.12	.10
4. change lanes less frequently while making/answering calls	.02	0.45	-.05	-.01	.04	.15	-.06	-.22	.11
5. shorten conversation while making/answering calls	.19	4.50 ^a^	-.10	.13	.03	-.10	.19	-.32 ^c^	-.11
6. remind the caller that he/she is driving	.10	2.04	.05	.18	-.01	-.07	.03	-.12	-.09
7. slow down when reading message	.15	3.14 ^b^	-.20	.03	.05	-.27	.45 ^a^	-.25	.19
8. pull over when reading message	.07	1.35	-.06	.03	.03	-.04	.04	.26	-.04
9. increase distance when reading message	.15	3.41 ^b^	-.20	.04	.02	-.42 ^b^	.43 ^b^	-.13	.21
10. change lanes less frequently when reading message	.17	3.95 ^b^	-.16	.06	.02	-.45 ^b^	.54 ^a^	.04	.07

CB means compensatory beliefs; SCB means safe compensatory beliefs.

^a^ *p* < 0.001;

^b^ *p* < 0.01;

^c^ *p* < 0.05.

### Drivers’ general compensatory beliefs and safe compensatory beliefs

The means and zero-order correlation coefficients for compensatory beliefs, mobile use activities while driving, and self-regulation behaviors are shown in Table 2. With regard to the results of correlations among demographic measures (i.e., age groups, gender, driving ages) and other measures, we found that age was significantly correlated with the following variables: general compensatory beliefs (i.e., substance use) (*r* = -0.27, *p* < 0.01), self-reported prior behaviors (i.e., making calls, sending short messages, slowing down/increasing distance/changing lanes less frequently when reading messages (*r* varied from -0.18 to -0.17, *p* < 0.05), and driving age (*r* = 0.28, *p* < 0.01). Gender was significantly correlated with shortened conversations (*r* = 0.18, *p* < 0.05) and reminding the caller while driving (*r* = 0.17, *p* < 0.01).

To address aim 1, the respondents’ self-reported compensatory beliefs of substance use and safe compensatory beliefs were analyzed. Overall, drivers reported positive ratings in terms of these two types of compensatory beliefs. As shown in the table, 17.1% and 23.6% of the respondents said that they had higher compensatory beliefs in terms of substance use and driving safety when using mobile phones, respectively. Consistent with this pattern, drivers had higher average response scores for safe compensatory beliefs (*M* = 3.14) than for general compensatory beliefs of substance use (*M* = 2.75), *t* (139) = 4.31, *p* < 0.001. The differences between demographic measures (i.e., gender, age groups, and driving experience) in the two types of compensatory beliefs were subjected to a multivariate ANOVA. Significant effects of age were found for general compensatory beliefs in substance use, *F* (1, 132) = 12.61, *p* < 0.01; the partial *η*^2^ was 0.09. The test showed that younger drivers reported greater substance use (*M*_30 or below_ = 3.16) than did members of the older age groups (*M*_31 or above_ = 2.35). A main effect of age was not found for compensatory safe beliefs. There were no significant main effects for gender or driving age, and there was no significant interaction between them.

As shown in Table 1, 19.3% and 20% of the respondents reported incidents or crash involvement caused by making/answering calls and sending/reading messages, respectively. A total of 26.4% of the respondents (n = 37) reported that they were involved in incidents or crashes caused by mobile use while driving. Although this proportion was small and not balanced with the proportion of those not involved in any incidents or accidents caused by mobile use while driving, we wanted to obtain an initial understanding of whether there was an influence of incidents or crash involvement on drivers’ self-reported compensatory beliefs. With this aim, the differences between drivers with incidents or accidents and safe drivers in the two types of compensatory beliefs were subjected to a multivariate ANOVA. Significant effects of this factor were found for general compensatory beliefs and safe compensatory beliefs, *F* (1, 138) ≧ 5.36, *p* < 0.05; the partial *η*^2^ varied from 0.04 to 0.08. The test showed that unsafe drivers held higher compensatory beliefs than did safe drivers (for substance use, *M*_unsafe drivers_ = 3.35 > *M*_safe drivers_ = 2.54; for safe compensatory beliefs, *M*_unsafe drivers_ = 3.60 > *M*_safe drivers_ = 2.98).

### Engagement in prior mobile phone use and self-regulatory behaviors

As shown in Table 2, in driving context, drivers tended to report less frequency of active mobile usage (i.e., sending text messages (17.14%) and making calls (23.57%)) than passive mobile usage (i.e., reading text messages (29.29%), answering calls (40.00%)). This pattern was consistent with drivers’ average response scores. The order of the reported frequency of mobile phone activities in prior behaviors from lowest to highest was (1) sending a text message (*M* = 2.51), (2) making a call (*M* = 2.92), (3) reading a text message (*M* = 3.24), and (4) and answering a call (*M* = 3.92). These findings were supported by t-tests, which showed the order of frequency to be answering > reading > making > sending for the driving context (*t* (139) ≧2.49, *p* < 0.05). Following this test, the differences between demographic measures (i.e., gender, age groups, and driving age) in the four self-reported usage variables were subjected to a multivariate ANOVA. Significant effects of age were found for active use (i.e., making calls and sending messages while driving), *F* (1, 132) ≧ 4.22, *p* < 0.05; the partial *η*^2^ varied from 0.03 to 0.04. The descriptive results showed that young and middle-aged respondents (*M*_making_ = 2.32, and *M*_answering_ = 2.55) reported engaging more frequently in these four usages than older respondents did. In particular, the difference in making calls (*M*_young_ = 3.35, and *M*_older_ = 2.67) and sending messages (*M*_young_ = 2.84, and *M*_older_ = 2.20) was significant. There were few differences between experienced and novice drivers of different driving ages regarding making and answering calls. Experienced drivers rated both making (*M*_novice_ = 2.73, and *M*_experienced_ = 3.29) and answering calls (*M*_novice_ = 3.72, and *M*_experienced_ = 4.30) significantly higher than novice drivers did, *F* (1, 132) = 4.11, *p* < 0.05, both partial *η*^2^ = 0.03. No other main effects or interaction effects were significant.

With respect to the involvement of self-regulatory behaviors, Table 3 shows that the majority of respondents reported that they engaged in regulated behaviors when using mobile phones while driving. In terms of the context of making or answering calls while driving, approximately 90% of respondents said that they reminded the caller that they were driving or shortened the conversation. With regard to regulated driving behaviors, more than 80% of the respondents tended to slow down or increase their distance; 77% of the drivers reported less frequency in changing lanes; and 60% said they pulled over more frequently. This pattern was consistent with drivers’ average response scores. The *t*-tests showed that there was no significant difference between the behaviors of reminding callers (*M* = 5.81) and conversation shortening (*M* = 5.75). Drivers tended to engage more frequently in these two behaviors than in most regulated driving behaviors (*M* ≦ 5.55; the difference between shortened conversations and slowing down was not significant) (*t* (139) ≧2.04, *p* < 0.05). With regard to the difference in engagement in prior regulated driving behaviors, *t*-tests showed the order of frequency to be slowing down (*M* = 5.55)/increasing distance (*M* = 5.44)/ changing lanes less frequently (*M* = 5.28) > pulling over (*M* = 4.51) (*t* (139) ≧4.47, *p* < 0.001) and slowing down > changing lanes less frequently (*t* (139) = 2.08, *p* < 0.05). The current study also surveyed these four driving regulated behaviors in the context of reading messages while driving. As shown in the table, 70%-77% of respondents tended to report changing lanes less frequently, increasing distance, and slowing down, and 51% reported pulling over when reading messages. Compared with making/answering calls, *t*-tests showed the same order of frequency: slowing down (*M* = 5.12)/increasing distance (*M* = 5.07)/changing lanes less frequently (*M* = 4.88) > pulling over (*M* = 4.25) (*t* (139) ≧3.87, *p* < 0.001), and slowing down > changing lanes less frequently (*t* (139) = 2.42, *p* < 0.05). The differences between the demographic variables (i.e., gender, age groups, and driving ages) in all twelve self-regulatory behaviors were subjected to a multivariate ANOVA. Significant effects of gender were found for reminding callers and shortening conversations during mobile conversations while driving, *F* (1, 327) ≧4.79, *p* < 0.05; all partial *η*^2^ were 0.04. The test showed that females reported engaging more frequently in these two conversation-related regulated behaviors (*M*_reminding_ = 6.05, and *M*_shortening_ = 6.06) than males (*M*_reminding_ = 5.62, and *M*_shortening_ = 5.57). No other significant main effects or interaction effects were found.

### Predicting mobile use while driving: the contribution of compensatory beliefs

Zero-order correlations and a series of hierarchical multiple regression analyses were used to analyze the relationships between mobile use behaviors and compensatory beliefs. As displayed in Table 2, general compensatory beliefs (i.e., substance use) and safe compensatory beliefs were all positively correlated with the main mobile usage (i.e., making calls, answering calls, sending messages, and reading messages). To test the predictive effects, a three-step hierarchical regression analysis was performed using demographic measures and compensatory belief variables. For each mobile use in prior driving contexts, the demographics (i.e., age group, gender, and driving age) were entered in Step 1, the safe compensatory beliefs were added in Step 2, and the general compensatory beliefs were added in Step 3. By controlling for the influence of other variables, this approach allowed us to assess the predictive utility of each type of compensatory belief. The results are summarized in Table 3. In Step 1, demographics were only able to explain 5.7% of the variance in making calls (*F*(3, 136) = 2.76, *p* < 0.05); the size of variance explained was smaller than 4.8% in other three mobile usages, and the regression models were not significant (*F*(3, 136) ≦ 2.31, *p* > 0.05). In Step 2, the safe compensatory beliefs, when added to the regression equations, were able to explain an additional 10.5%-29.3% of the variance in all prior mobile usage while driving, resulting in substantial and statistically significant increments to 15.3%-35.0% (*F*(1, 135) ≧ 16.70, *p* < 0.001). Safe compensatory beliefs emerged as a stronger predictor. In Step 3, the addition of the general compensatory beliefs to the regression model resulted in a substantial increase to 22.3%-47.1% of the variance in all prior mobile use behaviors accounted for (△*R*^2^ varied from 5.8% to 15.8%, *F*_*change*_ (1, 134) ≧ 11.74, *p* < 0.01), with general compensatory beliefs emerging as significant predictors in all prior mobile usage and safe compensatory beliefs emerging as significant predictors in making calls and reading messages while driving only. With respect to the contributions of demographics, driving age emerged as a significant predictor in both the sending and reading message regression model at Step 3. In summary, the contributions of compensatory beliefs were obvious, and general compensatory beliefs about substance use emerged as a very strong predictor for predicting frequent mobile usage behavior while driving. In the description section, we found that respondents reported more frequency of active mobile usage behavior than passive mobile usage behavior (i.e., making calls vs. answering calls and sending messages vs. reading messages). Here, as shown in Table 3, compensatory beliefs tended to be stronger predictors of active mobile use than passive mobile use, especially in the context of making or answering calls.

Following previous studies [18, 48, 49], a *t*-test method was used to compare the difference between unstandardized beta weights for variables of compensatory beliefs in active mobile use and passive mobile use [50]. With this approach, we can compare the predictive validity of significant predictor variables of compensatory beliefs across the two pairs of regression models (i.e., making calls vs. answering calls, sending messages vs. reading messages) in Step 2 and Step 3, respectively. In Step 2, compensatory safe beliefs were a significantly stronger predictor in making calls than in answering calls, *t* (270) = 2.17, *p* < 0.05, and the difference in this variable between sending messages and reading messages was not significant, *t* (270) = 0.05, *p* > 0.05. Using the third regression equation, the contribution of general compensatory beliefs was greater for sending messages than for reading messages, *t* (268) = 2.20, *p* < 0.05, although this significant difference was not supported statistically between making calls and answering calls, *t* (648) = 1.28, *p* < 0.05.

### Predicting self-regulatory behaviors: the contribution of compensatory beliefs

Aim 2 was to address the relationship between self-regulatory behaviors and compensatory beliefs as well as prior mobile usage behaviors in the context of driving. As shown in Table 2, compensatory beliefs (i.e., substance use and safe compensatory beliefs) were significantly correlated with prior self-regulatory behaviors of pulling over while using mobile phones, shortening conversations, and reminding callers that the driver is driving. A regression analysis was conducted to investigate the utility of predictors, especially compensatory beliefs and prior mobile use frequency in the driving context, for slowing down, pulling over, increasing distance, changing lanes less frequently, shortening conversation, and reminding the caller in corresponding use contexts (i.e., making/receiving calls and sending/reading messages). The results are shown in Table 4. As shown in the table, all measures, including demographic variables, explained a statistically significant portion of the variance in shortened conversation length, with general compensatory beliefs emerging as a significant predictor only. With respect to sending/reading message while driving, all variables collectively explained more than 15%-17% of the variance in slowing down, increasing distance, and changing lanes less frequently when reading message, with the prior frequency of sending or reading messages emerging as significant predictors only. In summary, the contributions of compensatory beliefs accounted for part of the variance in most prior self-regulatory behaviors. However, the predicting effect was not supported statistically, except in shortened conversation length.

## Discussion

In the current study, the main aim was to investigate drivers’ compensatory beliefs and their effects on mobile phone use while driving as well as self-regulatory actions. We identified the four most frequent mobile phone usages while driving and several self-regulatory actions for compensating for driving impairments. The results supported the efficacy of compensatory beliefs in predicting the primary behavioral activities (i.e., making calls, answering calls, texting and sending messages, and reading messages). However, the effects of compensatory beliefs were partially supported in explaining self-regulatory actions (i.e., shorten conversation length). Before illustrating these effects, we first discussed the degree of drivers’ compensatory beliefs as well as drivers’ prior mobile use activities and corresponding compensatory behavior engagement.

### Compensatory beliefs, mobile phone usage and corresponding self-regulation

In general, respondents reported lower or moderate degrees of compensatory beliefs, especially in response to general compensatory beliefs related to substance use (e.g., “Not drinking alcohol during the week can make up for the effects of drinking too much alcohol during the weekend”). The items related to substance use were initially used as a subscale to assess people’s compensatory healthy beliefs. Responses to this variable were considered a way to measure drivers’ general compensatory beliefs. We believe that drivers’ responses to the general compensatory beliefs were far from their responses to the topic of using mobile phones while driving. Therefore, if there is a relationship between general compensatory beliefs regarding substance use and mobile usage activities in the context of driving, the effects of compensatory beliefs on distracting mobile use would be supported. Compared with safe compensatory beliefs in terms of mobile use while driving, the majority of drivers in the current study reported lower compensatory beliefs. A significant difference between general compensatory beliefs and compensatory safe beliefs was found in this study. However, these two types of compensatory beliefs had a strongly correlated coefficient. Surprisingly, we found that safe drivers (i.e., those who reported no crashes or incidents caused by mobile use in prior driving experience) reported higher responses in relation to general compensatory beliefs and compensatory safety beliefs than unsafe drivers. The collective results found in the initial analysis indicated that it was appropriate to examine drivers’ compensatory beliefs to explain distracted driving.

In terms of prior mobile phone usage, the overall pattern of involvement in this study was consistent with previous studies [13]. Answering an incoming call was the most frequent mobile phone usage while driving, and texting and sending short messages was the least frequent usage. Unlike the previous findings in [13], the current survey found that the frequency of reading messages was higher than the frequency of making calls. The order of frequency was the same as the pattern of drivers’ perceived degree of distraction or risk, which is negatively related to risk-taking behaviors—that is, answering < reading < making < sending [16, 18]. In addition to understanding engagement in prior mobile phone use activities in terms of the perception of risk or distraction, the behaviors of answering calls and reading messages can be viewed as passive activities, whereas making calls and sending messages could be identified as active activities. We believe that this distinction should be further investigated.

With regard to drivers’ engagement in self-regulatory behaviors for mobile phone use while driving, the current study supported our expectation that drivers would like to adopt regulated actions to compensate for impairments in driving safety. Similar to previous studies [18, 19], the findings of the current study provide strong support for the frequent use of prior compensated actions at different levels. In accordance with Young et al.’s findings [10], self-regulatory behavior can occur at a strategic level (e.g., choosing not to use a mobile phone while driving), a tactical level (e.g., adjusting/regulating the timing of engagement), or an operational level (e.g., slowing down). In this study, 45% of respondents reported that they strategically chose not to answer an incoming call in their prior driving experience (*M* = 4.28). This result indicates that drivers tended to refuse calls. As mentioned above, we narrowed the self-regulatory actions within the scope of engagement in mobile phone use while driving. Compared with driving-related regulated behaviors at the operational control level, drivers tended to use conversation-related actions to maintain their driving within a safe margin at a tactical control level. This overall pattern was suitable for mobile phone use in the situations of making/answering calls and reading short messages while driving. This study presented a series of operational self-regulatory actions. The respondents tended to report greater frequency of slowing down/increasing distance from the car ahead, whereas they reported the least engagement in pulling over to the roadside. Again, these findings were consistent with previous study of [19] that showed that drivers can consciously use self-regulatory actions for driving stability when they are asked to assess their prior experience.

### The contributions of compensatory beliefs in predicting mobile use while driving

To test one of the main aims of the current study and examine the association between compensatory beliefs and mobile phone use while driving, hierarchical regressions were conducted to investigate the predictive effects of general and safe compensatory beliefs. This analysis attempted to explain why drivers want to be involved in conversations (i.e., making and answering calls) and engaging in short messages (i.e., sending and reading messages). Drivers’ personal characteristics, indicated by age group, gender, and prior driving experience, contributed less than 6% of the variance explained, from 15% to 47%. This finding suggests that personal characteristics are not a key determinant in predicting mobile phone use while driving, consistent with previous findings in [18]. Furthermore, the findings of the current study provide strong support for the ability of compensatory beliefs to predict the four types of mobile phone usage in the context of driving. The contribution of compensatory beliefs regarding driving were all very obvious for typical mobile phone usage. However, when general compensatory beliefs were included, safe compensatory beliefs emerged as significant predictors for explaining making calls and reading messages. The current study found that general compensatory beliefs, measured by six items of substance use, emerged as the strongest or core predictor of mobile phone usage while driving. In addition, it is surprising to note that general compensatory beliefs tended to be a stronger predictor of active use (i.e., making calls or texting and sending messages) than passive use (i.e., answering calls or reading messages).

As in our previous study in [18], which aimed to predict compensatory behavioral intentions to use mobile phones while driving within the TPB framework, the current study aimed to test the contributing effect of compensatory beliefs in predicting self-regulatory actions to compensate for negative performance caused by distracting mobile activities. The findings of this study partially supported this aim. Our expectations were supported only for prior behaviors of shortening conversations while using mobile phones in the context of driving. This means that the efficacy of the effect pattern of compensatory beliefs on self-regulatory actions was dependent on the influence of compensatory beliefs on mobile use behavior. Further studies could propose an integrated framework to illustrate the utility of compensatory beliefs in explaining why people use mobile phones and how they engage in corresponding compensatory actions.

These findings suggest that (1) the contribution of compensatory beliefs is strong in explaining why drivers use mobile phones even when they are aware of the risk; (2) compensatory beliefs involving distracted driving should be measured carefully; (3) the difference in the predicting effect between active and passive usages could be an interesting topic for further study; and (4) the associations among compensatory beliefs, mobile phone use and self-regulatory actions could be developed within a new predictive model. Previous studies have suggested that drivers’ behavioral beliefs, normative beliefs, and control beliefs, taken as a set in the TPB model, accounted for a high portion of the variance in mobile phone use while driving [12, 13, 18]. We also attempted to understand the contributions of mobile phone use and control beliefs in predicting drivers’ intentions to engage in compensatory actions within a modified TPB model [18]. We believe it would be helpful to combine compensatory beliefs with these three basic beliefs to understand the effects of compensatory beliefs on mobile phone use while driving as well as self-regulatory actions.

### Limitations of the current study

Several limitations should be considered when interpreting the current findings. First, the issue of how to identify and measure drivers’ compensatory beliefs should be tested. The items used for measuring drivers’ general compensatory beliefs in this study were suitable, and we believe that the compensatory beliefs that affect mobile phone use while driving are stable. However, compensatory beliefs were considered in the context of driving safety, and there is a lack of tools to assess drivers’ compensatory safety beliefs. Future studies could focus on developing a specific questionnaire to measure drivers’ compensatory safety beliefs. Second, similar to many previous TPB-related studies [13, 18, 37, 48], an important limitation of the present work is the reliance on self-reported behavioral engagements rather by questionnaire than actual behavior. Future research should address whether self-regulatory actions, especially at the operational level, are adopted by drivers in simulation experiments or field studies. Third, the findings regarding demographical measures’ impacts on respondents’ cell phone use and corresponding compensatory actions should be explained and clarified cautiously. We investigated the variables of age groups, gender, and driving ages in this study, and only found that age group emerged as a week significant predictor for predicting mobile phone use while driving. However, the result should be considered with noting the factor, i.e., 88.6% of all respondents in the present sample were highly educated with bachelor degree or above. This kind of high proportion was consistent with that appeared in other similar studies with using self-reported survey. By analyzing the responses of highly educated sample (i.e., 95% of respondents held degree of bachelor, master or above), Shi et al. succeeded in examining the factors affecting drivers’ choice to engage with a distracted mobile phone use in [16]. Combining all collective findings, we still believe that compensatory beliefs would be a significant predictor in explaining mobile phone use while driving with a sample of respondents with no college education in no college, but future study indeed need to address whether the respondents’ educational level bias the outcomes, especially in terms of other demographics’ influences on distracting mobile phone use.

Besides the limitations discussed above, the current study lacks an overall theoretical or conceptual construct for understanding the associations among compensatory beliefs, mobile phone use while driving and corresponding self-regulatory behaviors, though this study illustrated the predictive effects of compensatory beliefs in explaining why drivers use mobile phones while driving. In the regression analysis for self-regulatory actions, we found weak evidence to support these associations. We recognize that the impacts of compensatory beliefs on mobile phone use and self-regulatory actions may be complicated and need to be considered carefully with a systematical perspective. According to the illustrations for explaining how drivers compensate for distracted driving performance decrease in [10], environmental conditions were listed as one kind of important moderating factors in distracted driving contexts. We believe that road conditions would be a contributor for activating driver’s compensatory beliefs toward driving distractions, and this kind of variables should be considered in future study. For example, by combining the methods of survey and simulating experiment, researchers can test how traffic density at time of driving and the time of day (e.g., rush hour versus off-peak driving) moderates driver compensatory beliefs’ impact on their engagements in distracted activities. With understanding the moderating effects of driver’s characteristics and road environmental factors in distracted driving context, the associations among driver’s compensatory beliefs and distracted activities could be structured conceptually in future.

## Supporting Information

S1 Dataset (SAV)
